# T2 relaxation times of knee cartilage in 109 patients with knee pain and its association with disease characteristics

**DOI:** 10.1080/17453674.2021.1882131

**Published:** 2021-02-04

**Authors:** Joost Verschueren, Stephan J Van Langeveld, Jason L Dragoo, Sita M A Bierma-Zeinstra, Max Reijman, Garry E Gold, Edwin H G Oei

**Affiliations:** a Department of Orthopedic Surgery, Erasmus MC University Medical Center Rotterdam, The Netherlands;; b Department of Radiology & Nuclear Medicine, Erasmus MC University Medical Center Rotterdam, The Netherlands;;; c Department of Orthopedic Surgery, University of Colorado, Denver, CO, USA;; d Department of General Practice, Erasmus MC University Medical Center Rotterdam, The Netherlands;; e Department of Radiology, Stanford University Medical Center, CA, USA;; f Department of Bioengineering, Stanford University Medical Center, CA, USA;; g Department of Orthopedic Surgery, Stanford University Medical Center, CA, USA

## Abstract

Background and purpose — Quantitative T2 mapping MRI of cartilage has proven value for the assessment of early osteoarthritis changes in research. We evaluated knee cartilage T2 relaxation times in a clinical population with knee complaints and its association with patients and disease characteristics and clinical symptoms.

Patients and methods — In this cross-sectional study, T2 mapping knee scans of 109 patients with knee pain who were referred for an MRI by an orthopedic surgeon were collected. T2 relaxation times were calculated in 6 femoral and tibial regions of interest of full-thickness tibiofemoral cartilage. Its associations with age, sex, BMI, duration of complaints, disease onset (acute/chronic), and clinical symptoms were assessed with multivariate regression analysis. Subgroups were created of patients with abnormalities expected to cause predominantly medial or lateral tibiofemoral cartilage changes.

Results — T2 relaxation times increased statistically significantly with higher age and BMI. In patients with expected medial cartilage damage, the medial femoral T2 values were significantly higher than the lateral; in patients with expected lateral cartilage damage the lateral tibial T2 values were significantly higher. A traumatic onset of knee complaints was associated with an acute elevation. No significant association was found with clinical symptoms.

Interpretation — Our study demonstrates age, BMI, and type of injury-dependent T2 relaxation times and emphasizes the importance of acknowledging these variations when performing T2 mapping in a clinical population.

Knee osteoarthritis (OA) is currently mainly diagnosed on clinical presentation (Hunter and Bierma-Zeinstra 2019). Conventional radiography depicts morphological articular cartilage changes indirectly and is insensitive to both early-stage OA and subtle progression of the disease (Guermazi et al. 2011). MRI is able to visualize articular cartilage directly and is therefore more sensitive to osteoarthritic changes (Chan et al. 1991). But, similar to conventional radiography, conventional MRI relies primarily on the identification of morphological changes in damaged knee cartilage and is also limited to depicting relatively advanced signs of degeneration (McCauley et al. 2001). In the last 2 decades, innovative quantitative methods of MRI have been developed that have the potential to measure articular cartilage degeneration prior to morphological cartilage damage and, thus, might be able to identify cartilage at risk of developing irreversible cartilage damage (Matzat et al. 2013). A well-validated and quantitative MRI technique, transverse relaxation time (T2) mapping, is regarded as the best technique to determine the hydration content, collagen fiber orientation, and collagen network integrity in articular cartilage (Oei et al. 2014). These cartilage properties are known to be altered in the initial stages of OA development (Setton et al. 1999). T2 mapping is expressed in T2 relaxation times, which tend to increase with more advanced stages of cartilage damage (Dunn et al. 2004). The technique is widely used in scientific studies such as the Osteoarthritis Initiative (OAI) (Joseph et al. 2015). However, as current T2 mapping data is gathered in research settings with clear inclusion criteria based on age, sex, type of knee disorder, and OA stage, these results cannot directly be generalized to clinical practice (Joseph et al. 2015). Therefore, we assessed the association of T2 relaxation times of knee articular cartilage with patient and disease characteristics and clinical symptoms in an unselected routine clinical population of patients with knee complaints.

## Patients and methods

In a period of 18 months, all patients with complaints of knee pain referred for MRI of the knee by an orthopedic surgeon (JLD) from Stanford University Medical Center were eligible for the study.

### Image acquisition

The patients were scanned on a 3.0 Tesla (T) MRI scanner (MR 750, GE Healthcare, Milwaukee, WI, USA) with a flexible 16-channel receive-only coil (NeoCoil, Pewaukee, WI, USA). The patient’s knee was fixed with a leg holder in slight flexion to position the coil and reduce motion artifacts. In addition to a routine clinical knee MR protocol used by the radiologist to assess structural changes in the knee, a 3D fast spin echo T2 mapping sequence was added to the protocol during the trial period. This sequence with variable refocusing flip angle schedules uses T2 magnetization preparation followed by pseudo steady-state 3D FSE acquisition (Chen and Hat 2011, Matzat et al. 2015). The main T2 mapping sequence parameters were: 5 echo times (6, 12, 25, 38, 64 ms); 3 mm slice thickness; an in-plane resolution of 0.5 x 0.8 mm; and a scan time of approximately 6 minutes.

### Image analysis

**Figure 1.**
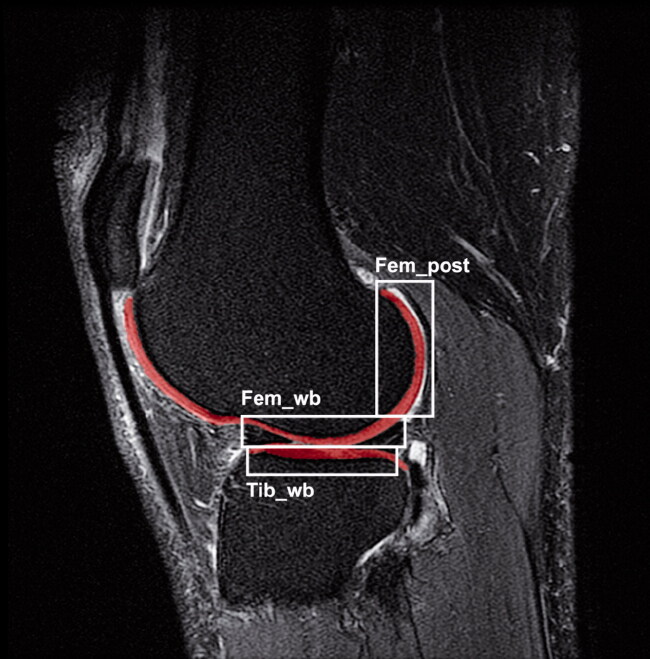
Cartilage segmentation on a T2-weighted image of the lateral compartment. Red area displays the femoral and tibial cartilage; white boxes represent the ROIs. Abbreviations: Fem_wb: weight-bearing femoral condyle; Fem_post: posterior femoral condyle; Tib_wb: weight-bearing tibial plateau.

The T2 mapping images were analyzed using an in-house developed MATLAB software tool (Bron et al. 2013). Full-thickness tibiofemoral cartilage masks were segmented on 6 slices (3 central slices of the medial and 3 central slices of the lateral compartment, respectively) of a sagittal T2 weighted sequence, which was part of the routine MR protocol (Figure 1). We used this sequence for segmentation because of better contrast between the cartilage and the surrounding tissue. The T2 mapping scan was subsequently registered to the T2 weighted scan using rigid registration to calculate T2 relaxation times in the segmented masks. The masks were further divided into a femoral weight-bearing, tibial weight-bearing, and femoral posterior region of interest (ROI) for both the medial and lateral knee compartment. The outer perimeters of the menisci demarcated the weight-bearing ROIs of the femur and tibia. The posterior ROIs contained the femoral cartilage behind the posterior border of the menisci. The 6 ROIs were also combined to calculate an average tibiofemoral T2 relaxation time for each knee.

### Patient and disease analysis

Patient characteristics (age, sex, and BMI), disease characteristics (diagnosis, duration of complaints, and onset of disease), and clinical symptoms were retrospectively collected through the electronic patient record. Diagnosis was based on the surgical report (when available), clinical report, and MRI report. The surgical report was considered the reference in case of discrepancies between the reports. The duration of complaints, defined as the period between the onset of knee pain and the date of the MRI, was divided into acute (< 1 month), subacute (1–6 months), and chronic (> 6 months). The onset of disease was specified as traumatic versus non-traumatic. To assess clinical symptoms, the Knee Injury and Osteoarthritis Outcome Score (KOOS) questionnaire was recorded for patients on their first visit to the Outpatient Clinic (Roos et al. 1998). In addition to the KOOS subscale (“symptoms’, “pain,” “activities of daily living,” “sport and recreation,” “quality of life”) scores, all 42 items of the KOOS were dichotomized into absence versus presence of knee complaints. When patients scored zero (i.e., no complaints), the complaint was considered absent, while a score of 1 to 4 indicated presence of the complaint. The KOOS questionnaire was disregarded when it was filled in more than 6 months before the MRI.

### Statistics

Statistical analysis was performed using SPSS (IBM SPSS Statistics for Windows, Version 21.0; IBM Corp, Armonk, NY, USA). Associations between T2 relaxation times and patient characteristics, disease characteristics, and clinical symptoms were tested using linear regression models. T2 relaxation times were used as dependent variable and patient characteristics, disease characteristics, and clinical symptoms as independent variables. We performed both univariate and multivariate analyses. Subgroups were created of patients with abnormalities expected to cause predominantly isolated medial (medial meniscal tear, medial bone marrow edema, or medial focal cartilage/osteochondral damage/degeneration) or lateral tibiofemoral cartilage changes (lateral meniscal tear, lateral bone marrow edema, or lateral focal cartilage/osteochondral damage/degeneration) (Su et al. 2013, Crema et al. 2014). When patients had abnormalities in both compartments of the knee, they were not included in the subgroups. Differences between the medial and lateral ROIs were tested with a paired t-test. Multiple imputations analysis was used for missing data. A p-value of less than 0.05 was considered statistically significant.

### Ethics, funding, data sharing, and potential conflicts of interest

The study was approved by the institutional review board of Stanford University Medical Center (protocol number 26840). Informed consent was obtained from all participants. This research was not supported by grants from any funding agency in the public, commercial, or not-for-profit sectors. The dataset that is necessary to replicate main findings can be obtained from the authors upon reasonable request. GEG and EHGO receive research support from GE Healthcare. The study was performed during the visiting professorship of EHGO at Stanford University Medical Center, which was partially funded by the Dutch Arthritis Foundation.

## Results

146 patients met the inclusion criteria of whom 109 were eligible for further analyses (Table 1). Main reasons for exclusion were no T2 mapping scan undertaken or insufficient quality of this scan due to metal and movement artifacts, which occurred relatively frequently because a surface coil was used instead of a dedicated knee coil. In 8 patients both knees were scanned. The most troublesome knee was included for analysis. The KOOS questionnaire was available for 55 subjects, as not all participants filled in the questionnaire at their first visit to the orthopedic surgeon. 8 questionnaires were disregarded because of the time interval with the MRI scan. No statistically significant differences were found in patient and disease characteristics between the patients with and without a KOOS questionnaire (data not reported).

**Table 1.** Population characteristics

Patient characteristics (n = 109)	
Male, n (%)	62 (57)
Age, years (SD)	41 (14)
(range)	(16–77)
BMI (SD)	26 (5)
Disease characteristics (n =109)	
Knee disorder causing medial tibiofemoral cartilage changes, n **^a^**	35
Medial meniscus injury	26
Medial bone marrow edema	6
Medial focal cartilage/osteochondral damage	9
Medial cartilage degeneration	4
Knee disorder causing lateral tibiofemoral cartilage changes, n **^a^**	21
Lateral meniscus injury	17
Lateral bone marrow edema	2
Lateral focal cartilage/osteochondral damage	5
Lateral cartilage degeneration	4
Duration of complaints, n (%)	
	18 (17)
1–6 months	22 (20)
	69 (63)
Onset of disease, n (%)	
Traumatic	47 (43)
Clinical symptoms (n = 47)	
KOOS subscales, score (0–100) (SD)	
Symptoms	63 (18)
Pain	45 (22)
Activities of daily living	66 (16)
Sports	74 (20)
Quality of life	35 (20)
T2 relaxation times (n = 109), ms (SD)	
Femoral and tibial cartilage	40 (3)
Weightbearing femoral condyle medial	41 (6)
Posterior femoral condyle medial	38 (4)
Weightbearing tibial plateau medial	40 (5)
Weightbearing femoral condyle lateral	40 (5)
Posterior femoral condyle lateral	37 (5)
Weight bearing tibial plateau lateral	41 (6)

a Patients can have more than 1 diagnosis.

### Patient characteristics

**Figure 2.**
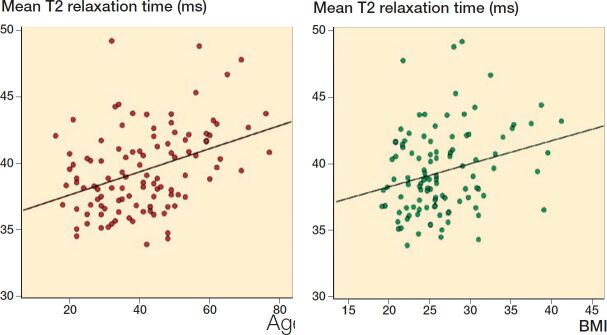
Scatter plots of age and mean T2 (left graph) and BMI and mean T2 (right graph) with corresponding trend lines (age: R^2^ = 0.15, and BMI: R^2^ = 0.068). Each circle represents the total tibiofemoral cartilage T2 value of 1 patient.

Data on BMI was missing for 3 patients. In the multivariate analysis with age, sex, and BMI as independent variables, age showed a statistically significant association with T2 relaxation times in all medial ROIs and the lateral weight-bearing tibial ROI, as well as the total tibiofemoral cartilage (Table 2). Increasing T2 relaxation times were seen with higher age. BMI showed a significant association with the total tibiofemoral cartilage. In the ROI analyses only, a significant association was seen in the lateral weight-bearing tibial cartilage. Sex did not seem to have an effect on T2 relaxation times. Figure 2 shows the scatter plots of age and BMI, respectively, with T2 relaxation times of the total tibiofemoral cartilage with the corresponding trend lines based on the (univariate) Pearson correlation coefficients.

**Table 2.** Multivariate linear regression of patient characteristics on total cartilage T2 values

	Age		BMI		Sex	
	β (95% CI)	p-value	β (95% CI)	p-value	β (95% CI)	p-value
Medial						
Femur weightbearing	0.34 (0.16 to 0.53)	< 0.01	–0.02 (–0.20 to 0.16)	0.8	0.02 (–0.17 to 0.20)	0.9
Femur posterior	0.09 (0.18 to 0.54)	< 0.01	0.07 (–0.11 to 0.25)	0.4	0.01 (–0.17 to 0.19)	1.0
Tibia weightbearing	0.26 (0.07 to 0.45)	0.01	0.07 (–0.11 to 0.26)	0.4	0.01 (–0.17 to 0.20)	0.9
Lateral						
Femur weightbearing	0.16 (–0.03 to 0.35)	0.1	0.14 (–0.05 to 0.33)	0.2	–0.41 (–0.23 to 0.15)	0.7
Femur posterior	0.00 (–0.20 to 0.19)	0.97	0.19 (–0.06 to 0.43)	0.1	0.04 (–0.15 to 0.23)	0.7
Tibia weightbearing	0.20 (0.02 to 0.38)	0.03	0.25 (0.07 to 0.44)	< 0.01	0.10 (–0.08 to 0.28)	0.3
Total	0.33 (0.16 to 0.51)	< 0.01	0.20 (0.02 to 0.38)	0.03	0.08 (–0.09 to 0.26)	0.4

Calculated coefficients are the standardized coefficients (β) with corresponding p-value and 95% confidence interval. In this model, the independent variables were responsible for 19% of the variance in T2 relaxation times (R^2^ = 0.19) and no multicollinearity was detected.

### Disease characteristics

We identified 35 patients with abnormalities that are the likely cause of medial cartilage damage. The medial femoral ROIs showed statistically significantly higher T2 relaxation times compared with the lateral femoral ROIs (Table 3). 21 patients were expected to have predominantly lateral cartilage damage. Statistically significantly higher T2 values were seen only in the lateral weight-bearing tibial ROI.

**Table 3.** Subgroups of patients with unicompartmental cartilage damage

Patients with	Medial	Lateral	p-value
	Mean T2 (SD)	Mean T2 (SD)	
Medial cartilage damage (n = 35)			
Femur weight-bearing	42 (9)	39 (4)	0.05
Femur posterior	37 (6	36 (4)	0.01
Tibia weight-bearing	40 (4)	40 (4)	0.5
Lateral cartilage damage (n = 21)			
Femur weight-bearing	41 (5)	39 (4	0.2
Femur posterior	37 (2)	37 (4)	1.0
Tibia weight-bearing	39 (3)	42 (5)	0.02

T2 values in milliseconds. Tested with paired sample t-test.

SD: standard deviation.

**Figure 3.**
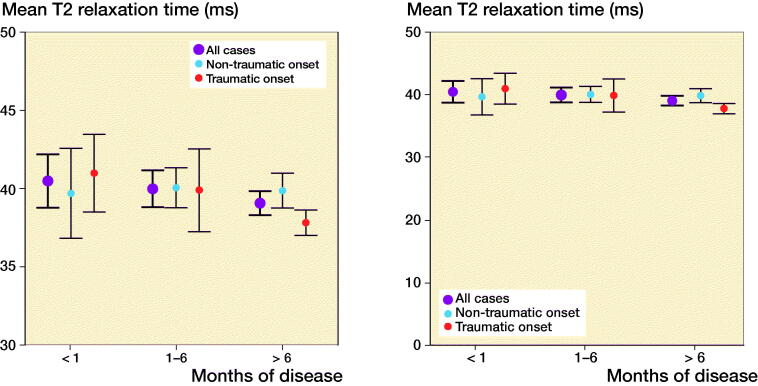
Total tibiofemoral cartilage T2 values with 95% confidence interval for duration of disease for all cases and divided in non-traumatic and traumatic onset groups classified as acute (n = 18 [7 and 11]), subacute (n = 22 [13 and 9]), and chronic (n = 69 [42 and 27]). Effect of duration on total cartilage T2 values for all cases was β = 0.31 (p = 0.4), for non-traumatic onset β = 0.06 (p = 0.6), and for traumatic onset β = –0.30 (p = 0.04) calculated by multiple linear regression analyses with sex, age, and BMI as covariates.

A trend towards decreased cartilage T2 values with an increase in duration of complaints was observed (Figure 3). However, this association was not statistically significant when the analysis was adjusted for age, sex, and BMI. In the case of a traumatic onset of knee pain, T2 relaxation times were the highest in patients with the shortest time between the onset and MRI acquisition. There was a gradual decline in T2 relaxation times between the MRIs undertaken in < 1 month, 1–6 months, and > 6 months after a traumatic onset. In patients with a non-traumatic onset of knee pain, T2 relaxation times appeared to be stable between the time points (Figure 3). These trends were seen for both the total tibiofemoral cartilage and the specific ROIs.

### Clinical symptoms

Mean KOOS values and standard deviations per subscale are displayed in Table 1. Univariate analyses showed a statistically significant association between clinical symptoms and total tibiofemoral T2 relaxation times for 2 of the 5 KOOS subscales (Pain: p = 0.02; Activities of daily living: p = 0.02). A lower score, i.e., more complaints, on the KOOS questionnaire was associated with elevated T2 relaxation times. When correcting for age, BMI, and sex, none of the associations remained significant. The item-specific analysis of the KOOS questionnaire revealed that, after adjusting for age, sex, and BMI, only “difficulties with descending stairs” was statistically significantly associated with elevated total tibiofemoral T2 relaxation times. Multivariate ROI-specific analysis did not show statistically significant associations with the different KOOS subscales either.

## Discussion

In this study, we assessed the association of T2 relaxation times of the tibiofemoral knee cartilage with patient and disease characteristics and clinical symptoms in an unselected clinical population of 109 patients. A positive statistically significant association was observed between T2 relaxation times and age and BMI, while sex did not have an effect on T2 relaxation times. Age seemed to have an overall effect on T2 values as increasing T2 values with increasing age were seen in most ROIs. Increasing T2 relaxation times with aging and higher BMI have previously been described in patients over 45 years old (Joseph et al. 2015). Furthermore, Mosher et al. found increasing T2 relaxation times in asymptomatic woman older than 45 compared with below 45 years (Mosher et al. 2004a). Our data shows these associations are seen in the whole adult range of age. BMI showed a trend towards increasing T2 values with increasing BMI, but a significant association was seen only in the lateral tibial weight-bearing cartilage. This is in contradiction to the findings of a recent paper that found an association between obesity and the risk of developing medial tibiofemoral OA (Wei et al. 2019). The range of T2 values in our study was between 35 and 50 ms, as can be seen in the scattorplots, which is in line with previously reported values (Oei et al. 2014). The increase in T2 relaxation time per unit of age or BMI was small, but this is what can be expected considering a difference of only 15 ms between the highest and lowest values. As most studies using T2 mapping focus on more advanced disease in selected patient groups, it is not surprising that larger differences in T2 values between damaged and healthy cartilage are found. We found no effect of sex on T2 relaxation times for both the total population and the age-dependent subgroups. A previously performed study looking at the influence of sex on T2 relaxation times also did not find such effect (Mosher et al. 2004b), but that study was based on a small and young population aged between 22 and 29 years. Other previous research showed only a weak association between T2 relaxation times and sex in the OAI population (age 45–65) without signs of radiographic OA (Joseph et al. 2015).

Differences in T2 values between medial and lateral compartments were found in patients with unicompartmental abnormalities. Previous studies with strict inclusion criteria already showed increasing T2 relaxation time in the medial knee compartment in patients with meniscal tears and in the lateral knee compartment in patients with anterior cruciate ligament injuries (Friedrich et al. 2009, Potter et al. 2012). Our study confirms this effect in a heterogeneous population. We found statistically significantly higher T2 values in the medial femoral ROIs in patients with abnormalities expected to cause predominantly medial cartilage changes. In patients with suspected isolated lateral cartilage changes, a statistically significant difference was found only in the tibial ROIs. Just like the correlations of age and BMI with T2 relaxation times, it is remarkable that higher medial femoral cartilage T2 values were associated with increasing age and medial abnormalities and higher lateral tibial values were associated with increasing BMI and lateral abnormalities. It would be interesting to assess the influence of mechanical leg axis on these findings, but as long leg radiographs were not available, it was not possible to answer this question.

Duration of complaints could potentially lead to transient variation of T2 relaxation times within patients as evidence is provided that the integrity of the cartilage collagen network is compromised soon after joint injury (Lohmander et al. 2003). Our study revealed higher T2 relaxation times in patients who had an interval of less than 1 month between trauma and MRI compared with patients with an interval longer than 6 months. However, since we did not perform follow-up measurements of the same patient, no conclusions regarding the trend over time of T2 relaxation time following trauma can be made based on our data. Nonetheless, it is worth noting that in the case of non-traumatic knee pain the duration of complaints did not cause variation in T2 relaxation times.

As far as we know, no imaging modality has shown a good correlation with clinical symptoms of knee injury and osteoarthritis in an unselected routine clinical population. In our study, significant associations were found between T2 values and two domains of the KOOS questionnaire in the univariate analysis. However, this finding was not sustained when corrected for age, sex, and BMI, with age being the predominant covariate. When looking at the item-specific analysis, we found only “any difficulty with descending stairs” to be correlated with T2 relaxation times after correction. Although a large set of symptoms was tested, and based on repeated testing coincidental findings are possible, previous studies also reported difficulties with climbing stairs to be a sensitive and prodromal symptom in osteoarthritis (Case et al. 2015, Landsmeer et al. 2019). The wide range in age and the known increase in knee complaints with age might be responsible for the absence of further associations between T2 relaxation times and clinical symptoms in our study (Paradowski et al. 2006).

Our study has several limitations. By using a clinical orthopedic population, we included patients with a wide range in age, BMI, diagnoses, and clinical symptoms. The combination of this heterogeneity and limited sample size could explain the absence of clear associations between T2 values and disease characteristics and clinical symptoms in our study. A second limitation is that we had a valid KOOS questionnaire available for only half of the patients. It was common practice at the Orthopedic Outpatient Department to ask patients to fill in the questionnaire. Unfortunately, this was not strictly controlled. We are aware that previous studies have shown T2 differences between superficial and deep cartilage layers (Mosher et al. 2004b, Bengtsson Moström et al. 2015). However, as our T2 mapping sequence is a 3D sequence with coverage of the whole knee, we considered the spatial resolution not good enough to perform these subregional analyses. Finally, we realize the magic angle effect could influence T2 values. However, as all patients were positioned in a standardized fashion, the effect would be similar for all patients. Together with the type of analyses we performed, we do not think the magic angle effect substantially influenced our results.

To date, the application of T2 mapping is primarily in clinical research with patient groups based on well-defined inclusion criteria. In contrast to the success of T2 mapping in research trials like the OAI, the poor associations of T2 mapping with patient and disease characteristics observed in our study illustrate the difficulties of implementing such a quantitative MR technique in a routine clinical population. In conclusion, our results emphasize the importance of acknowledging patient and disease characteristics when performing T2 mapping in a clinical population.

JV, SJvL, GEG, JLD, SMABZ, MR, and EHGO contributed to the design of the work. JV, SJvL, JLD, GEG, and EHGO contributed to the acquisition of the data. JV, SJvL, GEG, JLD, SMABZ, MR, and EHGO contributed to the analysis and interpretation of the data. All authors contributed to drafting the work or revising the content critically and all authors have approved the final version. JV and EHGO have full access to all of the data in the study and take responsibility for the integrity of the data and the accuracy of the data analysis.

*Acta* thanks Carl Johan Tiderius and other anonymous reviewers for help with peer review of this study.
